# The youth e-cigarette epidemic: New estimates of JUUL Labs’ revenue from youth users in the US

**DOI:** 10.18332/tid/133874

**Published:** 2021-04-29

**Authors:** Bekir Kaplan, Rajeev Cherukupalli, Kevin Welding, Ryan D. Kennedy, Joanna E. Cohen

**Affiliations:** 1 Institute for Global Tobacco Control, Johns Hopkins Bloomberg School of Public Health, Baltimore, United States

**Keywords:** JUUL, adolescent, electronic nicotine delivery systems

## Abstract

**INTRODUCTION:**

Past 30-day e-cigarette use increased by 78% among high school students from 2017 to 2018, an increase attributable to pod-style devices. JUUL Labs (JUUL) insists they do not market their product to teenagers. We created several scenarios to estimate the percentages of JUUL’s net revenue from adults and youth in the US in 2018.

**METHODS:**

We used the number of youth (aged 12–17 years) and adults (aged ≥18 years) who reported using JUUL in the nationally representative Population Assessment of Tobacco and Health (PATH) Study wave 4 (Dec 2016–Jan 2018) to estimate the youth proportion of JUUL users. As a sensitivity analysis, we also used data from the nationally representative Truth Longitudinal Cohort (TLC) study to estimate the youth proportion of JUUL users. Based on this percentage, we then applied several scenarios to estimate JUUL’s net revenue from youth in the US in 2018.

**RESULTS:**

From the PATH Study, 31% of JUUL users were youth (aged 12–17 years). In the TLC study, 30% of current JUUL users were aged 15–17 years. Given that JUUL’s net revenue was $1.3 billion in 2018, we calculated that JUUL made between $130 million and $650 million of its net revenue from youth, depending on consumption scenarios.

**CONCLUSIONS:**

A substantial proportion of JUUL’s profits in 2018 were a result of use by youth. It could be required that all e-cigarette companies actively ensure that use by youth is below a pre-determined small fraction of their sales, requiring that a high penalty be paid by those that fail to do so.

## INTRODUCTION

Current e-cigarette use increased by 78% among high school students, from 11.7% in 2017 to 20.8% in 2018, an increase attributable to pod-style devices such as those made by JUUL Labs (JUUL)^1^. Since being first marketed in 2015, JUUL became increasingly popular, accounting for nearly 75% of the market share in the US in 2018^2^. While JUUL insists that they do not market their products to teenagers^3^, evidence suggests that JUUL’s marketing clearly appealed to youth, following its entrance onto the market^4^. Two studies reported that youth obtain e-cigarettes from a variety of physical retailers, social sources such as friends or family, and online retailers^5,6^. To our knowledge, no study has estimated the amount of money JUUL has made from youth. Here, we estimate JUUL’s potential net revenue in 2018 attributable to use by youth in the US.

## METHODS

### JUUL’s revenue

JUUL’s 2018 domestic net revenue (i.e. its revenues from sales net of promotions and sales incentives like discounts) from devices and pods was $1.3 billion^7^. In 2017, 54% of JUUL’s revenue came from devices and 46% from pods^8^. Before applying the scenarios, we assumed that 54% ($702 million) of JUUL Labs’ 2018 revenue came from devices and 46% ($598 million) came from pods.

### Adult and youth JUUL users

We used the Population Assessment of Tobacco and Health (PATH) Study wave four (December 2016– Jan 2018) adult and youth data^9^. This dataset is a nationally representative study of tobacco use in the US able to provide information about JUUL use in both adults and youth.

In the PATH Study, ‘current use’ was defined as ‘respondents who have ever used any electronic nicotine products, have ever used them regularly, and currently use them every day or some days’ for adults, and ‘respondents who have used any electronic nicotine product within the past 30 days’ for youth. We used the PATH study team definition of adult current users^9^. In addition, current adult and youth users in this study stated ‘JUUL’ as the name of the brand they own. We combined youth and adult data to calculate the percentage of youth JUUL users. Youth do not always purchase their e-cigarettes themselves^5^, therefore, we also reported how youth users obtained JUUL. Analyses were performed using STATA version 15.1, incorporating the weights from the PATH Study for the prevalence estimates.

As a sensitivity analysis, we used data from Truth Longitudinal Cohort (TLC), a nationally representative study^10^ conducted between February and May 2018 to estimate the proportion of youth among JUUL users.

### Estimates of adult and youth JUUL sales

In total, 59 survey participants in the PATH Study stated ‘JUUL’ as the name of the e-cigarette brand they own. Of those JUUL users, 31.0% (95% CI: 18.3–47.2) (n=25) were youth (aged 12–17 years), 54.0% (95% CI: 40.0–67.3) (n=30) were young adults (aged 18–24 years), 1.8% (95% CI: 0.23–12.2) (n=1) were aged 25–34 years, and 13.2% (95% CI: 4.52–33.1) (n=3) were aged 35–44 years, after weighting.

Among 25 youth JUUL users in the PATH Study, 15.3% (n=4) got their JUUL by giving someone else money to buy for them, 23.5% (n=6) bought JUUL themselves from a store, 58.6% (n=14) bought from another person, and 2.6% (n=1) got a JUUL some other way.

The TLC study included 467 current JUUL users^10^, 29.1% (n=136) were aged 15–17 years, 57.2% (n=267) were aged 18–24 years, and 13.7% (n=64) were aged 25–34 years, which is very similar to the percentage of youth among JUUL users in the PATH data.

We created several scenarios to estimate the percentages of JUUL’s net revenue from adults and youth. These scenarios are based on 2017 reported revenue data from devices versus pods^8^.

JUUL use among youth might be under-reported because surveys may ask for e-cigarette use but not use the specific term ‘JUUL’ and youth might not realize JUUL is an e-cigarette^11^. On the other hand, a nationally representative study^12^ reported that 22.3% of past 30-day JUUL users aged 15–17 years used less than 1 pod over the period, suggesting that the intensity of JUUL pod use among youth could be lower compared to adults.

**Figure 1:**
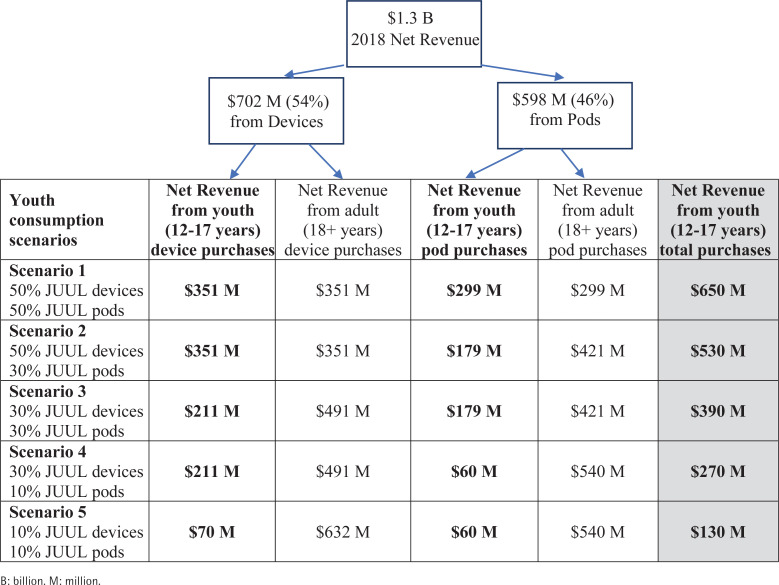
Different scenarios for calculating JUUL Labs’ net revenue from youth

Taking this into consideration, we created several scenarios with different assumptions about device and pod used by youth. The baseline scenario (Figure 1, Scenario 3) of 30% youth use of devices and pods relies on the youth proportion in both datasets. Other scenarios include: 1) Youth use 50% of JUUL devices and pods (most liberal scenario) (Figure 1, Scenario 1), a scenario assuming there is under-reporting of JUUL use among youth and that the intensity of pod use is similar for youth and adults; 2) Youth use 50% of JUUL devices and 30% of JUUL pods (Figure 1, Scenario 2), a scenario relying on assumption that rates of JUUL use are higher than reported but that pod use intensity by youth is lower than among adults; 3) Youth use 30% of JUUL devices and 10% of JUUL pods (Figure 1, Scenario 4), a scenario using the baseline device proportion and assuming that JUUL pod use is lower among youth than among adults; and 4) Youth use 10% of JUUL devices and pods (most conservative scenario) (Figure 1, Scenario 5), a scenario assuming youth users buy fewer devices (e.g. share devices) and fewer pods (lower intensity or non-JUUL pods) than their share of prevalence.

## RESULTS

Based on the scenario of 30% youth use of devices and pods, we calculated that JUUL made approximately $390 million from youth and $910 million from adults (Figure 1, Scenario 3). Based on our most conservative scenario, which assumes lower estimates of youth device and pod use, JUUL made $130 million, or 10% of its net revenue in 2018 from youth (Figure 1, Scenario 5). Our most liberal estimate, which adjusts for possible under-reporting of youth device and pod use, finds that JUUL’s potential net revenue from youth in 2018 was $650 million (Figure 1, Scenario 1), or 50% of the total net revenue. We also estimated that JUUL possibly made $530 million or $270 million from youth in 2018, based on two other less extreme under- and over-estimated scenarios of youth consumption (Figure 1, Scenarios 2 and 4).

## DISCUSSION

To our knowledge, this is the first study estimating JUUL’s revenue attributable to youth. We estimated that JUUL made between $130 million and $650 million of its net revenue in 2018 depending on youth consumption scenarios. This study found that in 2018 alone, JUUL made large and significant net revenues from users under the age of 18 years, in every scenario.

Tobacco control has advanced many policies to combat the youth e-cigarette epidemic, including prohibiting sales to youth, marketing restrictions, and flavor ban. One unexplored strategy is the explicit requirement, enforced through high penalties that e-cigarette companies actively ensure that youth use is below a pre-determined small fraction of sales. The regulatory intent and spirit of such a requirement would not be unprecedented. The Tobacco Masters Settlement Agreement^13^, for example, specifically prevented companies from actions that ‘initiate, maintain or increase the incidence of youth smoking’, and directed funding to reduce youth smoking. This may seem difficult to achieve, however, a similar situation for combustible cigarettes almost came into effect over two decades ago^14^. This could incentivize e-cigarette companies to minimize the use of their products among youth. In February 2020, the FDA announced they would prioritize enforcement against many flavored vape products appealing to youth, other than menthol and tobacco^15^. This may reduce the proportion of youth among JUUL users.

Consistent with the literature, most of the youth JUUL users (n=19; 76.5%) in this study got their product indirectly, but 24 out of 25 still noted paying money for their JUUL. In addition, 23.5% (n=6) of youth JUUL users reported that they directly bought JUUL from a store, suggesting that additional efforts are needed to increase compliance with current laws in the US prohibiting sales to minors.

### Limitations

The main limitation of this study is the small sample size of JUUL users in the PATH Study. We calculated the proportion of youth JUUL users from the TLC as a sensitivity analysis. To the best of our knowledge, these two datasets are the only nationally representative surveys to provide youth and adult data in the same survey. In the PATH Study, 19.2% (n=3) of adults were aged 35–44 years and 17.2% (n=4) of youth were aged 12–14 years. Given the similar percentages of those aged 12–14 years and 35–44 years, it is likely that if TLC included those age groups, the age distribution of the TLC Study would still be similar to the PATH Study. We could not adjust our results for price and intensity of e-cigarette use since the information was available for less than 20 out of 59 participants. Despite these limitations, using nationally representative data is a strength for this study.

## CONCLUSIONS

We estimate that JUUL potentially made between $130 million and $650 million (10–50% of its net revenue) in 2018 from underaged users. Quantifying the share of net revenue attributable to youth users can help hold e-cigarette companies accountable in the effort to minimize youth consumption of their products.
